# Supplementary material for: Portable, Rapid, and Sensitive Time-Resolved Fluorescence Immunochromatography for On-Site Detection of Dexamethasone in Milk and Pork
Source: Foods. 2021 Jun 10;10(6):1339. doi: 10.3390/foods10061339 (PMC8229924; doi:10.3390/foods10061339)
Supplement: Supplementary file 1 [file foods-10-01339-s001.zip › foods-1220407-supplementary.pdf]

Supplementary materials

Portable, rapid, and sensitive time-resolved fluorescence immunochromatography for on-site detection of dexamethasone in milk and pork

Xiangmei Li[1], Xiaomin Chen[1], Jinxiao Wu[2], Zhiwei Liu[1], Jin Wang[1], Cuiping Song[3], Sijun Zhao[3], Hongtao Lei[1,*], Yuanming Sun[1,*]

[1] Guangdong Provincial Key Laboratory of Food Quality and Safety, College of Food Science, South China Agricultural University, Guangzhou 510642, China

[2] Shanxi Institute of Feed and Veterinary Drug control, Taiyuan 030000, China

[3] China Animal Health and Epidemiology Center, Qingdao, Shandong 266032, China

* Corresponding author. Phone: +86 20 8528 3925. Fax: +86 20 8528 0270. E-mail: hongtao@scau.edu.cn, gzsyming@163.com

LC-MS/MS analysis

LC-MS/MS analysis was performed in multiple reaction monitoring (MRM) mode on a Shimadzu (Nexera x2, Japan)) LC system coupled with an AB Sciex triple quadrupole EMR (QTRAP®6500, USA). Chromatographic separation was performed on a Kinetex @ C18 column (100×3 mm, 2.6 μm), and the column temperature was maintained at 40 °C. The mobile phase was constituted of ultrapure water (mobile phase A) and acetonitrile (mobile phase B). The gradient elution procedure was as follows: 0~1.0 min, 20% B; 1.1~3.5 min, 50% B; 3.6~6.0 min, 65% B; 6.1~7.0 min, 20%~65% B; 7.1-7.5 min, 20% B. The mobile phase flow rate was 300 μL/min; the injection volume of the sample was 5 μL.

Mass spectra were obtained by an AB Sciex triple quadrupole EMR (QTRAP®6500, USA). The scanning mode is negative ion scanning (ESI-). The MS/MS conditions were set as follows: spray voltage: 5.5 KV; capillary temperature: 500 °C; data analysis software: Analyst 1.7.

Figure Caption

Figure S1 The results of particle size of TRFM

Figure S2 The fluorescence intensity, inhibition effect, and inhibition rate results of the activation pH value. A) Ultraviolet lamp results for milk detection; B) Fluorescence quantitative results for milk detection; C) Ultraviolet lamp results for pork detection; D) Fluorescence quantitative results for pork detection

Figure S3 The fluorescence intensity, inhibition effect, and inhibition rate results of the Ab amount. A) Ultraviolet lamp results for milk detection; B) Fluorescence quantitative results for milk detection; C) Ultraviolet lamp results for pork detection; D) Fluorescence quantitative results for pork detection

Figure S4 The fluorescence intensity, inhibition effect, and inhibition rate results of the surfactant concentration. A) Ultraviolet lamp results for milk detection; B) Ultraviolet lamp results for pork detection; C) Fluorescence quantitative results for milk detection; D) Fluorescence quantitative results for pork detection

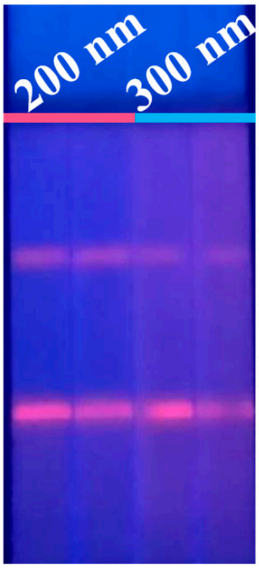

Figure S1

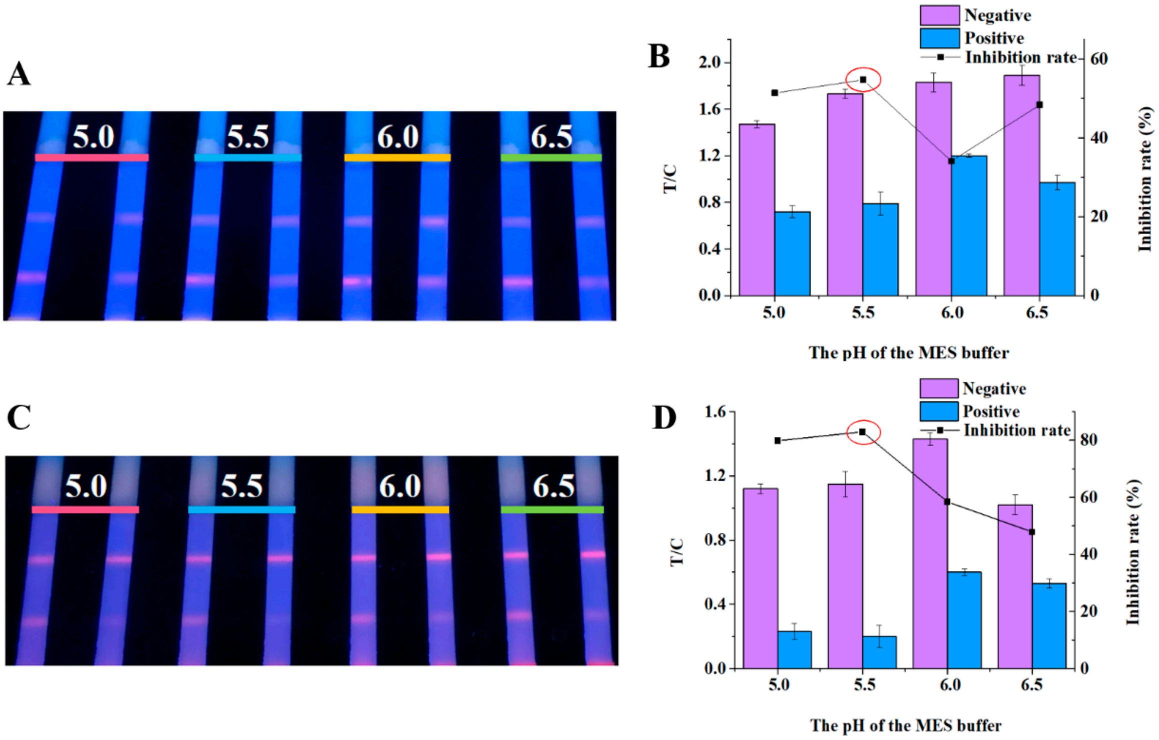
A
5.0
5.5
6.0
6.5
B
Negative
Positive
Inhibition rate
T/C
Inhibition rate (%)
The pH of the MES buffer
C
D

Figure S2

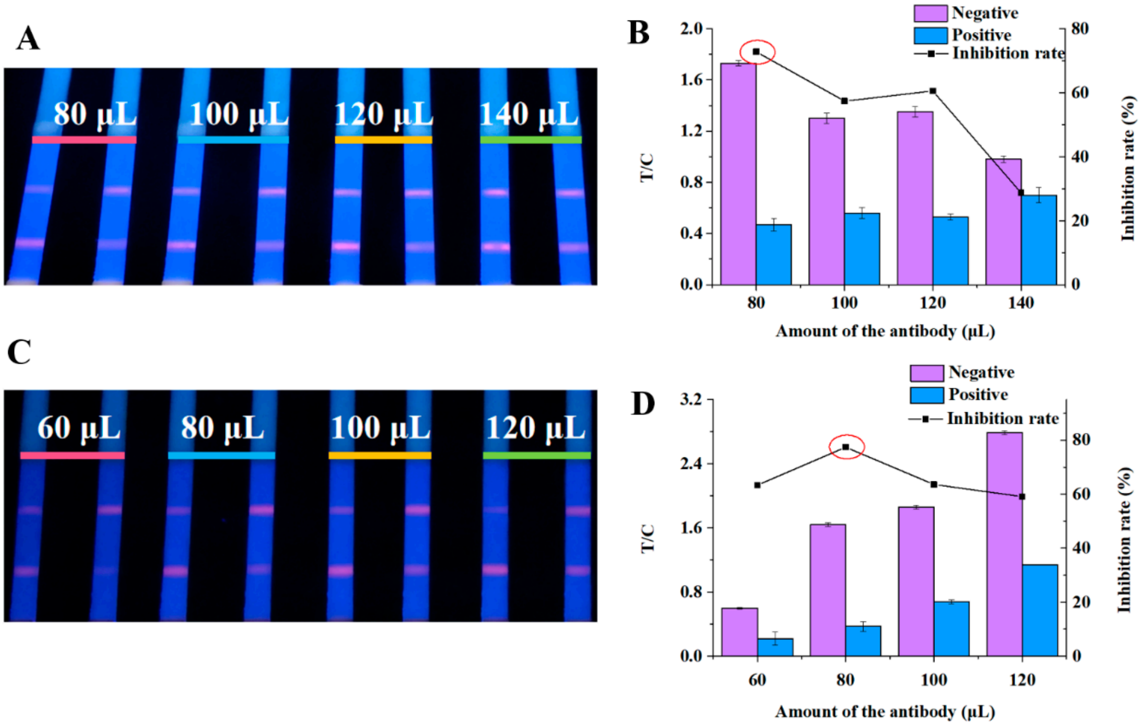

A
80 μL
100 μL
120 μL
140 μL
B
Negative
Positive
Inhibition rate
T/C
Inhibition rate (%)
Amount of the antibody (μL)
C
60 μL
80 μL
100 μL
120 μL
D
Negative
Positive
Inhibition rate
T/C
Inhibition rate (%)
Amount of the antibody (μL)

Figure S3

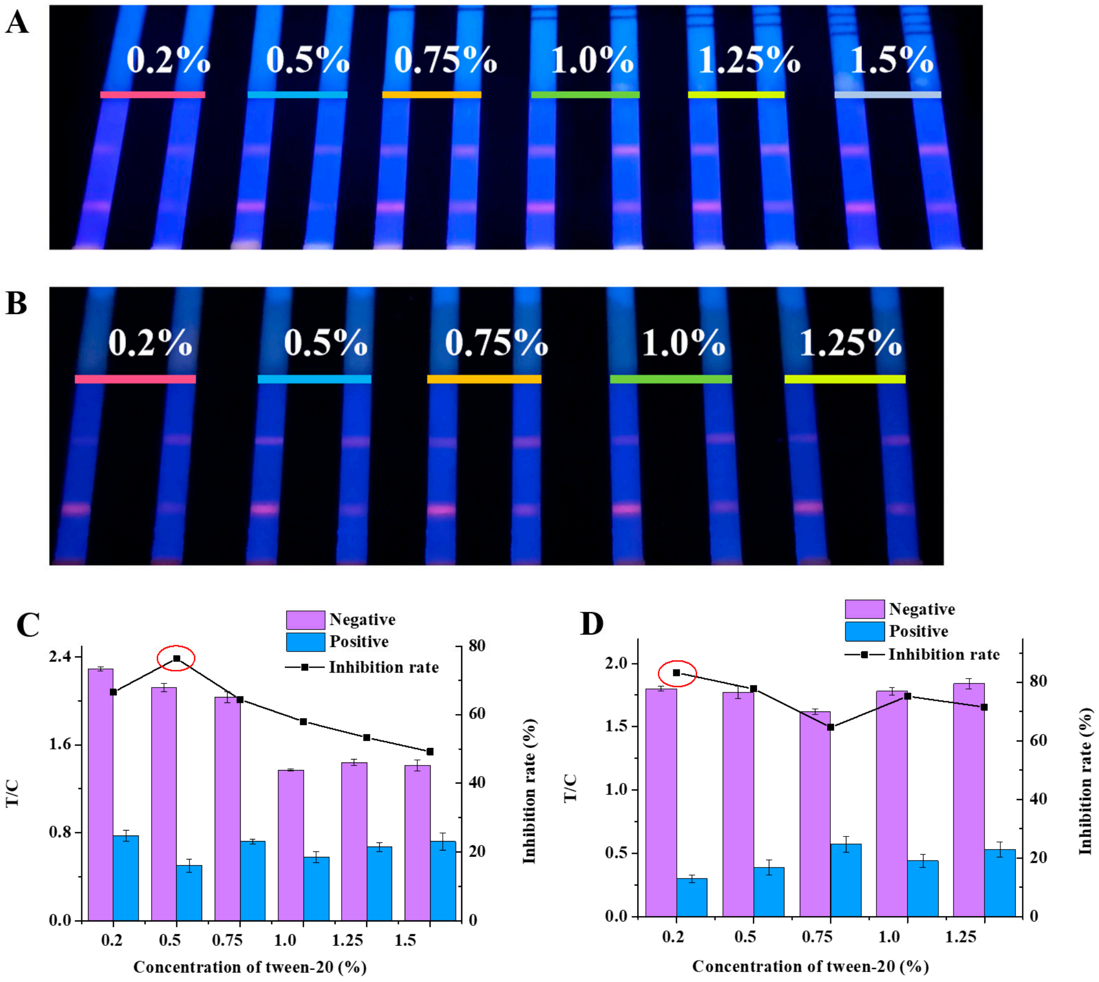
A
0.2%
0.5%
0.75%
1.0%
1.25%
1.5%
B
0.2%
0.5%
0.75%
1.0%
1.25%
C
Negative
Positive
Inhibition rate
T/C
Inhibition rate (%)
Concentration of tween-20 (%)
D
Negative
Positive
Inhibition rate
T/C
Inhibition rate (%)
Concentration of tween-20 (%)

Figure S4

Table S1 Working parameters of the TRFM-ICG for milk and pork detection

Working parameters	milk	pork
Particle size and color of nanoparticles	200 nm, red	
Concentration of coating Ag (mg/mL)	0.17	
Concentration of goat anti-mouse IgG (mg/mL)	0.22	0.44
Coating buffer	0.02 M PB (pH 7.4, 0.15 M NaCl)	
Activated pH value (0.05 M MES)	pH 5.5	
Coupling buffer	0.05 M BB (pH 8.0)	
Abs amount (mg/per strip)	1.5×10^{-5}	
Abs dilution buffer	0.002M BB (pH 8.0)	
Sample pad pretreatment solution	0.05 M PB (pH 7.4, 0.5% tween-20, 0.3% PVP)	0.05 M PB (pH 7.4, 0.2% tween-20, 0.3% PVP)
Abs probe resuspension	0.02 M PB (pH 7.4, 0.5% BSA, 0.5% tween-20, 5% trehalose, 0.2% PVP, and 0.03% procline-300)	
Sample pad material	GF-2	
Abs probe resuspension	0.02 M PB (pH 7.4, 0.5% BSA, 0.5% Tween-20, 5 % trehalose, 0.2 % PVP, and 0.03% procline-300)	

Table S2 The IC_{50} and CR values of anti-DEX Ab determined by icELISA, and TRFM-ICG

Analytes	icELISA		TRFM-ICG	
	IC_{50} (μg/L)	CR%	IC_{50} (μg/L)	CR%
Dexamethasone	0.06	100.0	0.05	100.0
Triamcinolone	0.11	54.5	0.08	62.5
Betamethasone	0.25	24.0	0.16	31.3
Prednisolone	0.43	14.0	0.24	20.8
Hydrocortisone	4.00	1.5	1.20	4.2
